# Novel somatic variants in CTNNA1 gene in Iranian patients with diffuse gastric cancer 

**Published:** 2021

**Authors:** Mohadeseh Naghi Vishteh, Tayyeb Ali Salmani, Amirreza Javadi Mamaghani, seyyed javad seyyed tabaei, Majid Kheirollahi

**Affiliations:** 1 *Department of Genetics and Molecular Biology, School of Medicine, Isfahan University of Medical Sciences, Isfahan, Iran*; 2 *Department of Medical Genetics, School of Medicine, Shahid Beheshti University of Medical Sciences, Tehran, Iran*; 3 *Department of Medical Parasitology and Mycology, School of Medicine, Student Research Committee, Shahid Beheshti University of Medical Sciences, Tehran, Iran*; 4 *Department of Parasitology, School of Medicine, Shahid Beheshti University of Medical Sciences, Tehran, Iran*

**Keywords:** Gastric cancer, CTNNA1, Mutation, Sequence analysis

## Abstract

**Aim:**

we aimed to evaluate somatic mutations of CTNNA1 in DGC patients.

**Background:**

Diffuse gastric cancer (DGC) is a major type of gastric cancer where most cases are sporadic diffuse gastric cancer (SDGC). It has been shown that mutations in CTNNA1 are responsible for some cases of hereditary diffuse gastric cancer (HDGC).

**Methods:**

In the present work, 48 formalin-fixed paraffin-embedded tissues, including samples of 38 SDGC and 10 HDGC patients were examined through Sanger sequencing approach on PCR products amplified from 18 exons and boundaries of intron/exon of CTNNA1 gene.

**Results:**

We revealed 9 novel somatic mutations in CTNNA1 gene in patients with HDGC and SDGC, from which one variant was intronic. Eight patients had at least one disease-causing mutation (16.6%). Most of the patients were in the III stage of cancer (50%). Except for one patient, histological type of the rest of mutation-harboring patients was signet ring cell carcinoma, and only one HDGC patient had CTNNA1 mutation.

**Conclusion:**

Our study showed several novel variants in the CTNNA1 gene in Iranian sporadic and hereditary DGC patients, and implies that the CTNNA1 gene mutations could be involved in the pathogenesis of DGC, either hereditary or in sporadic cases.

## Introduction

 Gastric cancer (GC) is the fifth prevalent type of cancer and the most recurrent cause of cancer-related deaths in the world after lung and bowel cancers (1, 2). GC has two main subtypes, diffuse GC (DGC, which is mostly undifferentiated) and intestinal-type GC (well-differentiated), which are distinguishable based on variable environmental aetiologies, clinical appearance, and genetic basis. As much as 90% of DGC cases are sporadic (SDGC), and hereditary DGC (HDGC) occurs approximately in 10% of cases. HDGC is a clinically distinct cancer predisposition syndrome, which has an autosomal dominant inheritance and commonly occurs in females and young patients (3). There is a familial predisposition in about 1–3% of gastric cancer patients. HDGC and SDGC are two morphologically similar entities of DGC, but they are distinguishable at the histological and immunohistochemical levels, and have different pathogeneses due to differential expression of CDX2 (4, 5). Previously, the International Gastric Cancer Linkage Consortium (IGCLC) defined the clinical criteria for every subtype of gastric cancer (6). The most common histological type of GC in the Iranian population was diffuse (59% and 56% in males and females, respectively), whereas the lowest prevalence belonged to the intestinal type of GC (7). From the histological view, the majority of GCs are multifocal and situated under a healthy mucosal layer. The tumor cells dose not form any mass and invade through adjacent tissues. Although the incidence of gastric cancer was decreased in recent years, the SRCC incidence increased continuously, in Asia, the United States and Europe, responsible for 35% to 45% of gastric cases (8).

To date, deleterious mutations in several genes including CDH1, CTNNA1(Catenin, Alpha 1), BRCA2, STK11, SDHB, PRSS1, ATM, MSR1, and PALB2 genes were found to be disease-causing in DGC (4, 9). It has been known that about 40% of families with HDGC have germline mutations in CDH1 and more than 100 diverse disease-causing germline mutations are described in families with different origins (2). CDH1 encodes the cell adhesion protein named E-cadherin, which comprises of a set of glycoproteins which play an important role in cell-cell adhesion and tight adherent junctions and thereby trigger cell differentiation and proliferation specificity of epithelial cells and invasion suppression (10, 11). The gene whose comatic mutations were evaluated in the present work namely CTNNA1, encodes α-catenin, binds the cytoplasmic domain of E-cadherin to the cytoskeleton via forming a complex with β-catenin, and is involved in cell-cell adhesion (12) as a result. The cadherin-catenin adhesion complex helps maintain epithelial tissue stability and dynamic cell movements during development and tissue renewal (13). It has been revealed that besides the CDH1 mutations, a germline truncating mutation in the a-E-catenin encoding gene CTNNA1 can cause HDGC, and the pattern of inheritance of this deleterious mutation in the evaluated family with 6 affected members was autosomal dominant. As mentioned above, CTNNA1 plays an important role in intercellular adhesion, being a suspected tumor suppressor in several cancers and a susceptibility gene for DGC (14). In the present study, we performed a clinical and genetic investigation and evaluated the mutational status of the CTNNA1 gene in 38 Iranian SDGC patients and 10 Iranian HDGC patients. Here, we report several novel CTNNA1 variants. 

## Methods

**Patients**

Our samples consist of 38 formalin-fixed paraffin-embedded (FFPE) tissue specimens from SDGC patients and 10 blocks of FFPE from HDGC patients collected and diagnosed in Taleqani Hospital, Shahid Beheshti University of Medical Sciences, Al-Zahra hospital, and Isfahan University of Medical Sciences, Iran, between 2007 and 2017. These cases were confirmed by a pathologist based on histopathological features and absent criteria based on International Gastric Cancer Linkage Consortium (IGCLC) (15). Informed written consent was taken from all the patients or their families. The study was approved by the Review Board of Isfahan University of Medical Sciences, in accordance with the ethical standards of the respective committee on human experimentation and with the Helsinki Declaration of 1975, as revised in 2000.

**DNA Extraction and Polymerase Chain Reaction (PCR)**

All Formalin-fixed paraffin-embedded (FFPE) samples were cut into 5-10 μm slices. DNA was extracted from FFPE sections using the One-4-All Genomic DNA Miniprep Kit (Bio Basic Inc., Canada). PCR was performed for all 18 exons and boundary intronic regions of CTNNA1 gene by a thermal cycler (Eppendorf AG, Germany) with 25 ml of reaction volume using the Taq DNA Polymerase Master Mix RED (Ampliqon, Denmark). PCR condition was as follows: the initial denaturation at 94˚C for 5 min, 33 cycles of 95˚C for 30 seconds, 55-60˚C for 30 seconds, 72˚C for 30 seconds and a final extension of 5 min at 72˚C. PCR primers were designed using primer 3 online primer design tool and listed in Table 1. To evaluate the presence of mutations in the germline, DNA was extracted from normal specimens (non-cancerous adjacent tissue) of all patients, and the presence of the detected mutation was tested in normal tissues.

**DNA Sequencing**

The products of PCR amplification then underwent direct sequencing using an ABI 3130XL capillary sequencing platform (Applied Biosystems/Life Technologies, Carlsbad, CA, USA). Sequence analysis of the CTNNA1 gene included all 18 exons and also the exon-intron boundaries. The results of sequencing obtained as electropherograms were compared to the reference sequence of the gene and analyzed with the Chromas software version 2.31. All detected variants were examined based on the local database (http://www.iranome.ir). Mutation Taster (http://www.mutationtaster.org) was used to evaluate sequence variants for their pathogenicity and disease-causing potential (16). Intronic variants were evaluated using Human Splice Finder (version 3.0).

**Table 1:** PCR primer sets for PCR and sequencing analysis in all 18 exons of CTNNA1

ID	sequence
CTNNA1 F1 ccttccctttccccaaaag CTNNA1 R1 cgacgtcgccaaagaaac CTNNA1 F2 cctgatgcaaaagtcccaaa CTNNA1 R2 agcagcgttctcaagggtta CTNNA1 F3 ttcagtggaatctcatgtg CTNNA1 R3 actgagcaattggcaagcat CTNNA1 F4 caaaggaacagcaaacaaca CTNNA1 R4 caaaaacatctctggtccattg CTNNA1 F5 aagggggagggaattttgta CTNNA1 R5 ttggcgaaggtaagtaatgg CTNNA1 F6 agaatcttacctaccaaacca CTNNA1 R6 gattcataatttcttttgact CTNNA1 F7 ggacagccccttacctgcta CTNNA1 R7 ctcccaaaaatcaaggagtca CTNNA1 F8 ccacataacacccctctgct CTNNA1 R8 ggcactttcttgtgaaaatcca CTNNA1 F9 tgaggggtcctcatgtaagtg CTNNA1 R9 tgttaagcgagcccttacaaat CTNNA1 F10 gtgcccttgtcatctgttcc CTNNA1 R10 tcccgtgcaaatgtcactta CTNNA1 F11 gcatgtggtgtgatgtctcc CTNNA1 R11 ctgccaagacatagcagtgtt CTNNA1 F12 tggcaccaagcaaacaataa CTNNA1 R12 ctcctccttcctcaccacag CTNNA1 F13 cccttcacacaggtagaagca CTNNA1 R13 aatatactgccaccgcatctg CTNNA1 F14 aagcagaggctcgagacaaa CTNNA1 R14 gagagagagaaacggtgttctga CTNNA1 F15 aatagcccttcaggcgaaat CTNNA1 R15 gcccctcacagttggagtag CTNNA1 F16 actttcttccccacaggtca CTNNA1 R16 gcccatgaaacttaccctga CTNNA1 F17 cacactgaacctttcagaaacag CTNNA1 R17 cggataggaggtgactttca CTNNA1 F18 tagggggctccccttcaa CTNNA1 R18 cccatttccctgtggaatct	

## Results

**Epidemiological and clinicopathologic results**

In general, 48 DGC patients (32 males and 16 females) were evaluated, aged 29–83 years, of whom 38 patients were SDGC and 10 were affected with the hereditary type of DGC (HDGC). The mean age of the patients at diagnosis was 56.73 years (57.37 and 50.3 for male and female patients, respectively). In 11 diagnosed patients (22.9%) , the tumor was at early TNM stages (I, II), and in 25 cases (52%) it had been recognized in late stages (III, IV). Also, the TNM stage of the tumor had not been determined in 12 patients (25%). Based on the histopathological evaluations, the tumor type in 39 cases (81.2%) was “Signet ring cell carcinoma” and in 9 (18.8%) cases, the histopathological type was evaluated as “ poorly differentiated adenocarcinoma” (Table 2).

**Sequence analysis**

All the coding exons and intronic boundary regions of the CTNNA1 gene were successfully amplified using PCR in all patients, and DNA sequencing of the amplified PCR products showed 10 variants in the CTNNA1 gene of the tumor samples, among which one synonymous alteration was reported previously (Table 3). There were three synonymous exonic alterations, and the evaluation of an intronic variant revealed a potential effect on the splicing site in one variant. Among the 48 patients, 10 patients were identified separately with at least one heterozygous CTNNA1 variant. Except for one, these variants were absent in the local population database (http://www.iranome.ir) and were not found in the disease databases (Clin Var, OMIM, HGMD, and literature in PubMed till to Jul 29, 2018). Seven variants were predicted as disease-causing by Mutation Taster. After examining the presence of detected mutations in the normal tissue of affected patients, it had been revealed that the mutation in exon 10 of samples ID 1 and 7, as well as the mutation detected in exon 16 of sample ID 37, was formed in the normal tissue of the patient (Table 3). This implies that these mutations are raised from germline.

**Amino acid and intronic substitutions**

**Figure 1:**
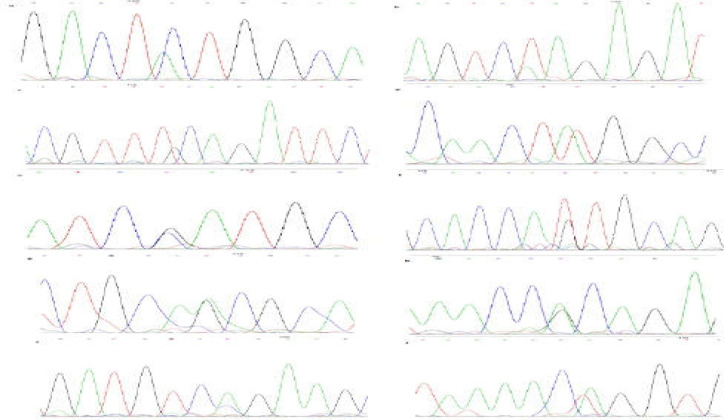
Sequence electropherogram of the CTNNA1 gene. The location of the base substitutions is shown by the arrow. (a) Sequence electropherogram of the exon 2 CTNNA1 gene (c.67C>A). (b) Sequence electropherogram of the exon 2 of the CTNNA1 gene (c.53T>A). (c) Sequence electropherogram of the exon 9 of the CTNNA1 gene (c.1171T>G). (d) Sequence electropherogram of the exon 10 of the CTNNA1 gene (c.1307T>A). (e) Sequence electropherogram of the exon 13 of the CTNNA1 gene (c.1837G>C). (f) Sequence electropherogram of the exon 16 of the CTNNA1 gene (c.2288T>G). (g) Sequence electropherogram of the exon 17 of the CTNNA1 gene (c.2343A>G). (h) Sequence electropherogram of the exon 11 of the CTNNA1 gene (c.1425A>G). (i) Sequence electropherogram of the exon 14 of the CTNNA1 gene (c.1951A>C). (j) Sequence electropherogram of the exon/intron boundary 4 of the CTNNA1 gene (c. 300A>T).

Six single base-pair substitutions in 10 samples were detected including a single-base pair substitution (c.67C>A) in exon 2 in three samples, a single-base-pair substitution (c.53T>A) in the same exon in two samples, a single-base-pair substitution in exon 9 in one sample (c.1171T>G), a single-base-pair substitution (c.1307T>A) in exon 10 in two samples, one single-base-pair substitution (c.1837G>C) in exon 13 in three samples, and lastly, a single-base-pair substitution (c.2288T>G) in one sample, all resulting in single amino acid substitutions (Fig. 1a-f). Figure 1g showed a synonymous benign variant (c.2343A>G) that was discovered in exon 17 of the gene in two samples. Also, two single-base-pair substitutions (c.1425A>G and c.1951A>C) were detected which did not change to the amino acid at a corresponding (Fig. 1h and i). 

**Table 2:** Epidemiologic and clinicopathologic features of sporadic diffuse gastric cancer patients

Gender	Age of diagnosis	Stage	Histopathological type	Sporadic or Hereditary
Female Male Male Male Male Male Male Female Male Male Male Male Male Female Male Male Male Male Male Female Male Male Male Male Female Female Male Male Female Male Female Female Female Male Male Male Male Female Female Female Male Female Female Male Male Male Female Male	*51* *54* *75* *73* *78* *65* *31* *61* *53* *80* *51* *54* *62* *29* *40* *53* *60* *64* *39* *73* *60* *57* *66* *31* *51* *40* *67* *70* *65* *50* *32* *74* *66* *58* *57* *62* *61* *37* *68* *65* *49* *55* *63* *73* *52* *40* *40* *51*	I II III IV III II III III III - III III III III III II I - III - IV III III - - - - III IV IV - - III III - III II II II III III II IV II - - II III	Signet ring cell carcinoma Signet ring cell carcinoma Signet ring cell carcinoma Signet ring cell carcinoma Signet ring cell carcinoma Signet ring cell carcinoma Signet ring cell carcinoma Poorly differentiated denocarcinoma Signet ring cell carcinoma Poorly differentiated denocarcinoma Poorly differentiated denocarcinoma Signet ring cell carcinoma Signet ring cell carcinoma Signet ring cell carcinoma Signet ring cell carcinoma Signet ring cell carcinoma Signet ring cell carcinoma Signet ring cell carcinoma Signet ring cell carcinoma Signet ring cell carcinoma Signet ring cell carcinoma Signet ring cell carcinoma Poorly differentiated denocarcinoma Poorly differentiated denocarcinoma Signet ring cell carcinoma Signet ring cell carcinoma Poorly differentiated denocarcinoma Signet ring cell carcinoma Poorly differentiated denocarcinoma Poorly differentiated denocarcinoma Signet ring cell carcinoma Signet ring cell carcinoma Poorly differentiated denocarcinoma Signet ring cell carcinoma Signet ring cell carcinoma Signet ring cell carcinoma Signet ring cell carcinoma Signet ring cell carcinoma Signet ring cell carcinoma Signet ring cell carcinoma Signet ring cell carcinoma Signet ring cell carcinoma Signet ring cell carcinoma Signet ring cell carcinoma Signet ring cell carcinoma Signet ring cell carcinoma Signet ring cell carcinoma Signet ring cell carcinoma	S S S S S S H S S S S S S H H S S S H S S S S H S H S S S S H S S S S S S H S S S S S S S H H S

As mentioned above and shown in Figure 1j, a single-base-pair substitution in the intronic boundary of exon 4 of the CTNNA1 gene was found in three samples (c. 300A>T). Bioinformatics analysis using the Human Splicing Finder showed that this alteration may be affected by the splicing site, and new potential splice sites could be created (Table 4).

**Table 3:** Exonic and intronic variants in the CTNNA1 gene in patients with sporadic and hereditary diffuse gastric cancer

Sample ID	substitution	Exon or exon/intron boundary	Homozygosity	Amino acid	Chromosome location	Prediction
9, 13, 17	C>A	2	Heterozygot*	L23M	NM_001903: c.67C>A	disease causing
9, 17	T>A	2	Heterozygot*	L18Q	NM_001903: c.53T>A	disease causing
5, 9, 7	A > T	4	Heterozygot*	Intronic	NM_001903: c. 300A>T	disease causing
9	T>G	9	Heterozygot*	S391A	NM_001903: c.1171T>G	disease causing
1, 7	T>A	10	Heterozygot*	L436*	NM_001903: c.1307T>A	disease causing
1, 9, 10, 19	A>G	11	Heterozygot*	no AA changes		-
13, 37, 42	G>C	13	Heterozygot*	D613H	NM_001903: c.1837G>C	disease causing
1, 13	A>C	14	Heterozygot*	no AA changes	NM_001903: c.1951A>C	-
37	T>G	16	Heterozygot*	I763S	NM_001903: c.2288T>G	disease causing
9, 13	A>G	17	Heterozygot	no AA changes		Benign

**Table 4:** The intronic variant which affects splicing site

Consensus value (0–100)	Splice site type	variant	Sample ID
75.14	Acceptor	NM_001903: c. 300A>T	5, 9, 7

Consensus value: splice site if ≥ 65

## Discussion

As we know GC is the fifth most prevalent neoplasm worldwide, accounting for 8.3% of all cancer-related mortality in 2018 (17). Generally, the vast majority of GCs are sporadic and only 10% of patients have a positive family history, of whom 1–3% are affected with the hereditary type of GC (18, 19). About 30% of GC patients belong to diffuse-type gastric cancer (DGC) or poorly-differentiated histological type, characterized by a lack of intercellular adhesion and the presence of scattered signet-ring cell morphology (20, 21). GC has a wide-spread global incidence, without any known reason, and its manifesting features are different in low- and high-risk regions. (22). An epidemiological study by Almasi et al. showed that unlike the global statistics, GC is the most deadly type of cancer in Iran, especially in the Northern regions. Also, they stated that over the past 30 years, the GC incidence has increased in our country (23). To date, deleterious mutations in several genes including CDH1, CTNNA1, BRCA2, STK11, SDHB, PRSS1, ATM, MSR1, and PALB2 genes were found to be disease-causing in DGC (9).

To evaluate somatic mutations of the CTNNA1 gene, we sequenced 48 tissue samples acquired from hereditary or somatic GC patients using a Sanger sequencing approach. In this study, several somatic mutations in the CTNNA1 gene were reported in patients with HDGC and SDGC, of which eight variants were novel. From among 48 patients, disease-causing mutations were found in ten. Most patients were in stage III of cancer (50%). Except for one patient, the histological type of the rest of the patients was signet ring cell carcinoma. Only one patient with the disease-causing mutation was affected with HDGC. In a young patient (31 years old) with HDGC, we found an intronic variant in exon 4 and a single-base-pair substitution in exon 10 (resulting in the amino acid substitution), where both mutations appear to be disease-causing after analysis with various bioinformatics tools. Interestingly, only one benign synonymous-mutation variant was found. Finally, two new variants were found with unknown significance (VUS).

The first report that supposed a predisposing role for CTNNA1 in GC was performed by Majewski et al. on a large Dutch HDGC family with no obvious mutation in CDH1 using exome sequencing. They found a germline truncating variant of CTNNA1 due to a 2bp deletion in exon 2 of CTNNA1, which results in a frameshift after Arg27 (p.Arg27Thr.fs*17). They also detected the mutation in the somatic tissue of patients (14). In another study, Hansford et al. revealed two novel truncating germline mutations in CTNNA1 (N71fs and R129X) in two unrelated families (2). In 2017, Weren et al. detected two germline frameshift variants including p.Asn443fs and p.Arg330fs in CTNNA1 in two unrelated GC cases. They confirmed that inactivating mutations in CTNNA1 is an infrequent cause of DGC predisposition (24). Altogether to date, only five confirmed variants in the CTNNA1 gene in patients with GC have been reported. We reported 9 novel variants, mostly in SDGC patients.

Majewski et al. also found that the remaining CTNNA1 allele was silenced in diffuse gastric cancers which implies the crucial role of this gene in the pathogenesis of diffuse gastric cancer (14). As we know, germline mutations in CDH1 are responsible for 30–40% of hereditary diffuse gastric cancers (HDGCs) (2). Like CDH1, CTNNA1 is involved in intercellular adhesion and is a suspected tumor suppressor and susceptibility gene for DGC (14). It has been suggested that the pathogenicity underpinning GC susceptibility in CDH1 mutation carriers is transmitted through a CDH1/CTNNA-1 signaling axis (2).

In recent years, Waren et al. sequenced a large cohort of 283 patients with CDH1-negative, familial GC using a targeted next-generation sequencing approach. They found numerous truncating germline variants in CTNNA1, confirming that loss-of-function mutations in CTNNA1 trigger the development of (familial) HDGC (24). As we know, CTNNA1 gene encodes α-E-catenin, which functions in the same junctional complex as E-cadherin, encoded by the main HDGC predisposing gene CDH1 (24). In another recent research on individuals with gastric or breast cancer, it had been revealed that there are several loss-of-function (LOF) and missense variants in the CTNNA1 gene. They concluded that the overall risk of gastric cancer for CTNNA1 LOF carriers may be lower than expected (25).

In conclusion, our study reveals several novel variants in the CTNNA1 gene in Iranian patients with both sporadic and hereditary DGC. Our data is in concordance with the theory that the CTNNA1 gene mutations could be involved in the pathogenesis of DGC, either hereditary or in sporadic cases.
